# Successful thrombolysis of portal vein thrombosis induced by post-liver transplant splenectomy: a case report

**DOI:** 10.3389/frtra.2025.1689539

**Published:** 2025-10-16

**Authors:** Xu Yan, Pusen Wang, Yiming Huang, Dong Zhao, Lin Zhong

**Affiliations:** ^1^ Department of Liver Surgery and Organ Transplantation Center, Shenzhen Third People’s Hospital, Second Affiliated Hospital, Southern University of Science and Technology, Shenzhen, Guangdong, China; ^2^ Department of Surgery, Graduate School of Medicine, Kyoto University, Kyoto, Japan

**Keywords:** liver transplantation, splenectomy, portal vein thrombosis, thrombolysis, anticoagulation

## Abstract

**Introduction and importance:**

Liver transplantation (LT) is a life-saving procedure for patients with end-stage liver disease, but post-transplant complications, such as portal vein thrombosis (PVT), can significantly impact patient outcomes. PVT is particularly challenging when it occurs after splenectomy, which is sometimes necessary in LT recipients with persistent hypersplenism or thrombocytopenia. The optimal management of PVT in this context remains unclear, and further clinical insights are needed.

**Case presentation:**

We present a case of a 57-year-old male with a history of chronic hepatitis B-induced liver cirrhosis who underwent LT. Due to persistent hypersplenism and thrombocytopenia, the patient later underwent splenectomy. One month post-splenectomy, the patient developed PVT, which was initially managed with anticoagulation therapy (aspirin and rivaroxaban). Despite treatment, thrombosis progressed, requiring intravenous heparin and urokinase thrombolysis. Serial imaging confirmed thrombus resolution, and the patient was discharged on long-term anticoagulation therapy.

**Clinical discussion:**

PVT following splenectomy in LT patients is a complex and potentially life-threatening condition influenced by altered portal hemodynamics and a hypercoagulable state. The standard treatment involves anticoagulation, but there is no consensus on the optimal regimen in post-transplant patients. This case highlights the potential efficacy of peripheral urokinase infusion as an alternative to interventional thrombolysis, particularly for patients who refuse invasive procedures. Long-term anticoagulation and close monitoring are crucial to prevent recurrence.

**Conclusion:**

This case underscores the importance of early detection, tailored anticoagulation strategies, and a multidisciplinary approach in managing PVT following splenectomy in LT recipients. Peripheral urokinase infusion may serve as a viable treatment option for patients with contraindications or reluctance toward invasive procedures. Further studies are needed to optimize anticoagulation protocols and long-term management strategies in this patient population.

## Introduction

Liver transplantation (LT) has emerged as the most effective treatment for end-stage liver cirrhosis, significantly enhancing patient survival rates and improving their quality of life. In liver transplant recipients, splenectomy may be performed in cases of persistent hypersplenism, thrombocytopenia, or splenomegaly that do not respond to less invasive interventions such as splenic artery embolization. Hypersplenism, characterized by the overactive destruction of blood cells within the spleen, can exacerbate the risk of bleeding and infection post-transplantation (1).

Nevertheless, postoperative complications remain a major concern, with portal vein thrombosis (PVT) being one of the most severe and potentially life-threatening issues (2). PVT is a severe and potentially life-threatening complication that can occur after liver transplantation, with or without additional procedures like splenectomy. The incidence of PVT in liver transplant recipients varies, but studies suggest that it can occur in number of cases, depending on various risk factors, including the patient's underlying liver disease, the presence of cirrhosis, and the complexity of the surgical procedure (3). The pathophysiology of PVT involves a combination of altered portal hemodynamics, endothelial injury, and a hypercoagulable state, which can be exacerbated by splenectomy (4). Splenectomy increases platelet counts and can induce a hypercoagulable state, thereby heightening the risk of thrombosis in the portal venous system. PVT can compromise graft function and patient outcomes; therefore, prompt diagnosis and intervention are imperative (5, 6). The management of PVT typically involves anticoagulation therapy, with heparin, oral anticoagulants, and vitamin K antagonists being the first-line treatments (7). However, there is no consensus on the optimal anticoagulation regimen, particularly in the context of LT. In some cases, thrombolysis or surgical intervention may be necessary, especially when PVT is complicated by splenectomy (8). The prognosis of PVT largely depends on the promptness of diagnosis and the effectiveness of the therapeutic strategy employed. This case report has been reported in accordance with the SCARE (Surgical Case Report) criteria (9).

## Case presentation

### Patient background

**Figure 1:**
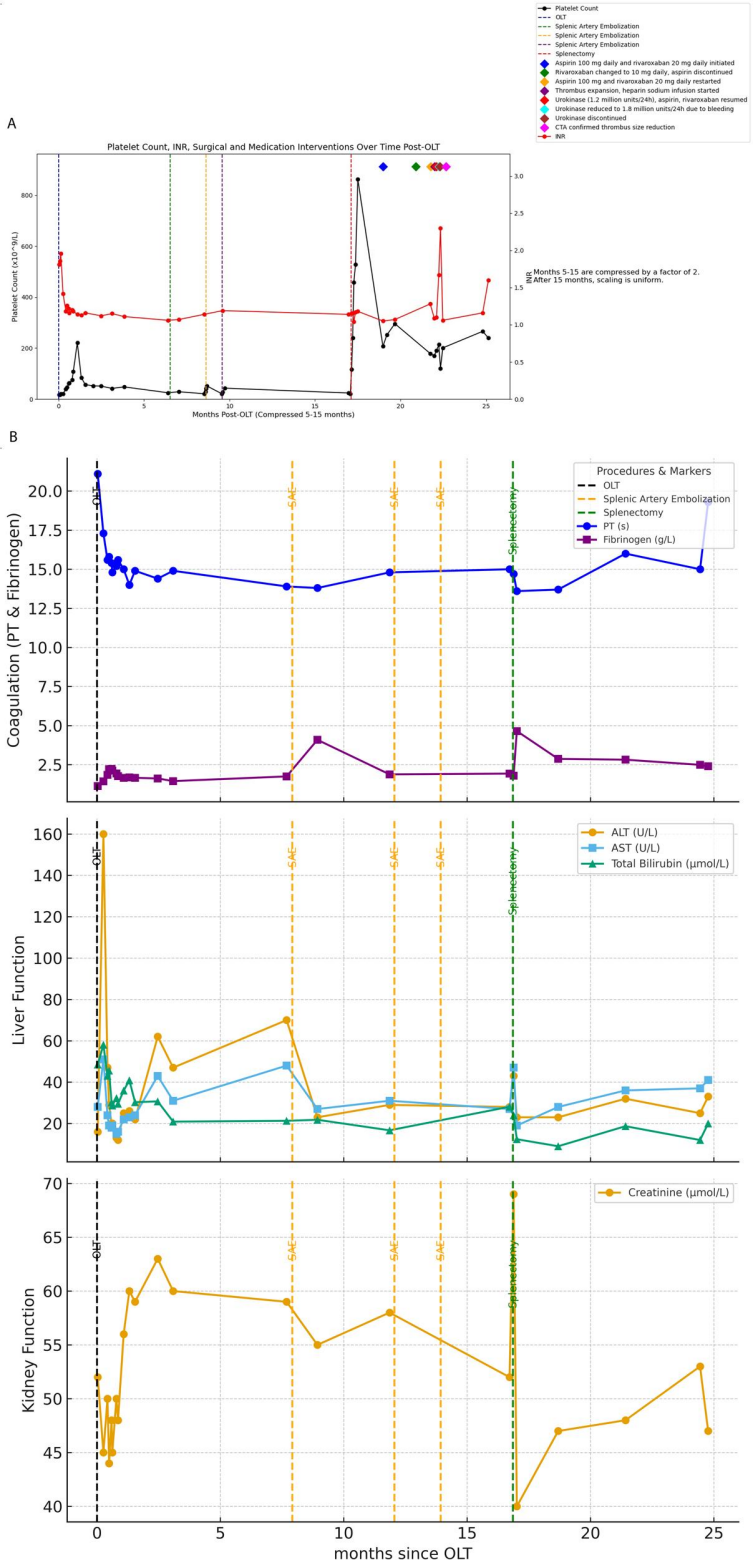
**(A)** Shows all surgical procedures and the timing of anticoagulant administration following the patient's liver transplantation, along with a line graph depicting the changes in platelet count and INR over the corresponding time period. **(B)** Illustrates the changes in liver function, coagulation function, and creatinine throughout the entire treatment course from OLT to splenectomy.

A 57-year-old male with a medical history of chronic hepatitis B-induced liver cirrhosis, hypersplenism, and esophageal and gastric varices was admitted for orthotopic LT due to the aforementioned conditions. Pre-transplant evaluation showed a MELD score of 17, total bilirubin 48.5 μmol/L, AST 28 U/L, ALT 16 U/L, white blood cell count 1.15 × 10⁹/L, hemoglobin 101 g/L, and platelet count 18 × 10⁹/L. HBV serology revealed HBsAg 47.6 IU/ml, HBsAb negative, HBeAg negative, HBeAb positive, and HBcAb positive. The patient underwent piggyback liver transplantation. The surgical procedure took place on August 30, 2022 (The liver donors in this study was brain-dead donors. Organ procurement was conducted only after obtaining informed consent or authorization from the donors' families. No organs from executed prisoners were used in this study). Postoperative immunosuppressive therapy included tacrolimus prolonged-release capsules (4 mg/day) and mycophenolate mofetil (1 g every 12 h). In addition, the patient received propofol tenofovir for antiviral therapy. Following transplantation, there was notable improvement in liver function (Figure 1B); however, splenomegaly and hypersplenism persisted, and platelet levels remained consistently low. Within one year after OLT, the platelet count was still around 20 × 10⁹/L.

### Intervention

The patient underwent splenic artery embolization on April 18, 2023, August 22, 2023, and October 18, 2023 due to persistent hypersplenism and thrombocytopenia; however, these interventions failed to ameliorate the condition of hypersplenism or improve platelet counts. On January 15, 2024, the patient underwent splenectomy under general anesthesia. Postoperative platelet levels initially exhibited improvement (Figure 1A).

#### Complications

**Figure 2:**
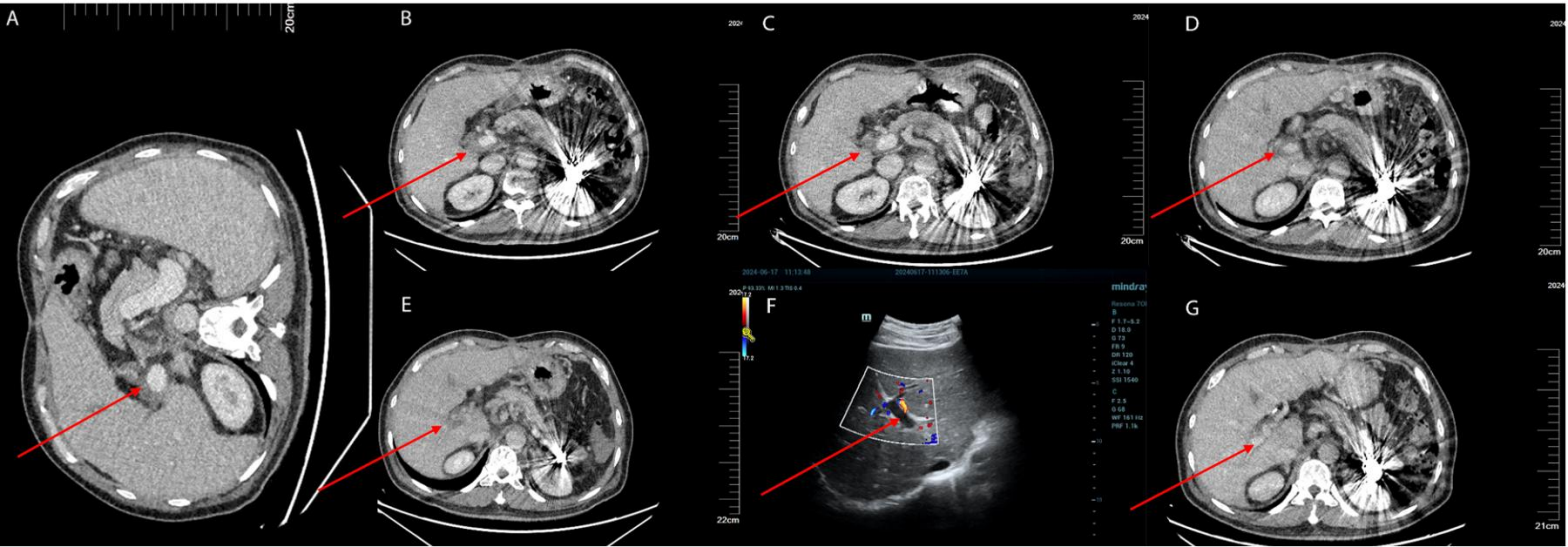
**(A)** The patient underwent a follow-up enhanced abdominal CT scan on January 11, 2024, prior to splenectomy, which showed no thrombus in the portal vein. **(B)** The patient underwent an enhanced abdominal CT scan on March 11, 2024, with the red arrows indicating the location of the portal vein thrombosis. **(C)** The patient had a follow-up enhanced abdominal CT scan on April 2, 2024, which showed that the extent of the thrombosis had reduced compared to the previous scan. The red arrows indicate the location of the portal vein thrombosis. **(D)** The patient underwent a follow-up enhanced abdominal CT scan on June 3, 2024, which revealed a new thrombus in the right branch of the portal vein. The red arrows indicate the location of the portal vein thrombosis. **(E)** The patient had a follow-up enhanced abdominal CT scan on June 10, 2024, which showed an increase in the extent of the portal vein thrombosis compared to the previous scan. The red arrows indicate the location of the portal vein thrombosis. **(F)** The patient underwent a follow-up liver Doppler ultrasound on June 17, 2024, which still showed the presence of a portal vein thrombosis. The thrombus had not completely occluded the portal vein. The red arrows indicate the location of the portal vein thrombosis. **(G)** The patient underwent a follow-up enhanced abdominal CT scan on June 30, 2024, which demonstrated a decrease in the size of the portal vein thrombus compared to the previous scan. The red arrows indicate the location of the portal vein thrombosis.

Despite initial improvements, the patient discontinued antiplatelet therapy (aspirin 100 mg daily and clopidogrel 75 mg daily) one month after surgery. Subsequent liver artery CTA revealed thrombosis (Figure 2B). On March 11, 2024, the patient was initiated on aspirin 100 mg daily and rivaroxaban 20 mg daily for anticoagulation. Follow-up CTA on April 2, 2024 demonstrated a reduction in thrombus size (Figure 2C).

### Further complications and treatment

Due to the extraction of an inflamed wisdom tooth, the patient's medication was changed to rivaroxaban 10 mg once daily and aspirin was discontinued. On May 8, 2024, a PVT was detected through ultrasound in local hospital. The anticoagulation regimen was adjusted to aspirin 100 mg once daily and rivaroxaban 20 mg once daily. On June 3, 2024, a follow-up CTA revealed a new small thrombus in the right branch of the portal vein (Figure 2D). Intravenous infusion of heparin sodium was initiated while oral anticoagulants were ceased. A subsequent CTA on June 10, 2024 demonstrated an expansion of the thrombus (Figure 2E). Because the patient had previously undergone multiple splenic artery embolization procedures, he refused further interventional treatment at this stage. Therefore, peripheral vein infusion of urokinase was selected as the alternative approach. On June 13, 2024, peripheral infusion of urokinase (1.2 million units per 24 h) commenced along with aspirin at a dose of 100 mg once daily and rivaroxaban at a dose of 20 mg once daily.

The patient presented with gingival bleeding and hematuria, leading to a reduction in urokinase dosage to 1.8 million units per 24 h. On June 17, 2024, liver ultrasound revealed significant improvement in portal vein thrombosis (Figure 2F). Urokinase infusion was discontinued on June 19, 2024.

### Outcome

A CTA on June 30, 2024 confirmed a decrease in thrombus size, indicating successful treatment (Figure 2G). The patient was discharged with continued oral administration of aspirin at a dose of 100 mg daily and rivaroxaban at a dose of 20 mg daily.

(The image displays the timeline of medication interventions, illustrating the specific periods during which each treatment was administered and the timing of changes in the medication regimen. The connected lines represent the duration of each treatment before a new intervention was introduced).

## Discussion

The management of PVT in post-liver transplantation patients presents a formidable and multifaceted challenge, particularly when complicated by additional procedures such as splenectomy (10). This case underscores several key points in the diagnosis, treatment, and management of PVT in this patient population.

1. Pathophysiology and Risk Factors:

Portal vein thrombosis (PVT) in liver transplant patients can arise due to multiple factors. The primary risk factors include changes in portal hemodynamics, endothelial injury, and the hypercoagulable state often seen post-surgery (5). Splenectomy further exacerbates these risks by increasing platelet counts and potentially contributing to a hypercoagulable state (11). However, the etiology remains unknown in 30%–40% of patients (12). In this patient, persistent hypersplenism and low platelet counts post-transplant necessitated splenic artery embolization and eventual splenectomy, setting the stage for thrombotic complications.

2. Impact on liver function:

Typically, mild portal vein thrombosis (PVT) does not severely impact liver function unless the patient has cirrhosis (5). In this case, the patient's liver function tests were closely monitored, Liver function remained stable, with normal transaminase and bilirubin. Although most of the PVT dissolved, the patient's portal vein blood flow remained low. Therefore, effective management of PVT is crucial to prevent deterioration of liver function, which is vital for the overall prognosis of liver transplant recipients.

3. Treatment Strategies:

The first-line treatment for portal vein thrombosis (PVT) is anticoagulation therapy, which mainly includes heparin, oral anticoagulants, and vitamin K antagonists (7). Currently, there is no consensus on the optimal anticoagulation regimen for PVT (13). Initial management involved anticoagulation with aspirin and rivaroxaban. Studies have shown that rivaroxaban has comparable anticoagulant effects to warfarin but with a lower risk of bleeding (14). The combined use of antiplatelet and anticoagulant drugs is common in patients with atrial fibrillation and those undergoing coronary interventions, though it significantly increases the risk of bleeding (15). In PVT treatment, the combined use of antiplatelet and anticoagulant therapy is less common. However, the patient's decision to discontinue antiplatelet therapy contributed to the development of PVT. Although discontinuation of antiplatelet therapy likely contributed to thrombus progression, it is noteworthy that the patient also developed a new PVT while still receiving anticoagulation. This suggests that additional factors may have been involved. One possible explanation is an underlying thrombophilia or other prothrombotic condition, which could predispose to recurrent thrombosis despite therapy. In this case, routine thrombophilia screening was not performed, which is a limitation. Future cases should consider systematic evaluation for inherited or acquired thrombophilia when PVT occurs under adequate anticoagulation. For acute PVT, mesenteric artery infusion of urokinase is often chosen (16). Although the reduction of anticoagulation therapy contributed to thrombus progression in this patient, it is also important to note that a new PVT developed despite ongoing therapy. This suggests that additional underlying factors may have played a role. One possible explanation is thrombophilia or other prothrombotic conditions, which can predispose patients to recurrent thrombosis even under anticoagulation. In this case, routine screening for inherited or acquired thrombophilia was not performed, which represents a limitation. Future cases should consider evaluating for such conditions, especially when thrombotic events occur despite adequate anticoagulation.

During the urokinase infusion, The patient developed gum bleeding and hematuria, without gastrointestinal bleeding. Coagulation tests showed low fibrinogen, which improved after dose reduction. For patients who refuse interventional treatment, peripheral urokinase infusion can effectively control thrombus but carries a bleeding risk. Continuous monitoring of coagulation levels and adjusting the infusion dosage are necessary. The switch from oral anticoagulants to intravenous heparin, and later to urokinase infusion, reflects the need for aggressive management in the face of an expanding thrombus. At the same time, our case should be considered in the context of previously reported cases and reviews. Several reports have linked splenectomy performed at or after liver transplantation with a higher risk of *de novo* portal vein thrombosis (PVT). In adult living donor liver transplantation, splenectomy was independently associated with early and late PVT, with hazard ratios around 3–5 in multivariable analysis (4). Similar observations have been made in other cohorts, where splenectomy during liver transplantation exposed recipients to a higher thrombosis rate, and thus careful surveillance of portal vein patency is recommended (17). Major societies (AASLD, EASL, Baveno VII) advise anticoagulation for non-tumoral PVT when bleeding risk is acceptable and recommend center-specific pathways for intervention (18). Overall, these data underscore that PVT prevention and management after LT—and especially post-splenectomy—requires individualized decision-making, balancing thrombotic and bleeding risks.

4. Outcome and Follow-Up:

The resolution of PVT was confirmed by serial imaging, demonstrating the effectiveness of the chosen therapeutic approach. Long-term anticoagulation with aspirin and rivaroxaban was maintained to prevent recurrence especially in the patient with splenectomy. This case highlights the importance of long-term follow-up and the potential need for ongoing anticoagulation in patients with a history of PVT.

5. Lessons Learned:

Patient Education: Ensuring patients understand the importance of adherence to anticoagulant therapy is crucial. Discontinuation of therapy without medical advice can lead to serious complications.

Monitoring and Early Intervention: Regular monitoring and early intervention at the first sign of thrombotic complications can significantly improve outcomes.

Multidisciplinary Approach: Managing PVT requires a multidisciplinary approach involving hepatologists, surgeons, radiologists, and hematologists to provide comprehensive care.

Long-term Anticoagulation Consideration: Splenectomy after liver transplantation is associated with a higher risk of portal venous system thrombosis. Therefore, long-term prophylactic anticoagulation should be considered in selected patients, with individualized risk–benefit assessment and close follow-up, as suggested by recent reports and reviews (3, 7, 10, 11).

This case underscores the need for continued vigilance and a proactive approach in the postoperative management of liver transplant recipients, particularly those undergoing additional procedures such as splenectomy. Future research should focus on optimizing anticoagulation strategies and developing standardized protocols to improve the management and prognosis of PVT in this patient population.

6. Limitation:

This case report has several limitations. First, long-term follow-up data after discharge were not available, as the patient did not continue surveillance at our center. Therefore, we cannot provide further information on portal vein patency, recurrence of thrombosis, or long-term clinical outcomes. Second, routine screening for inherited or acquired thrombophilia was not performed. As the patient developed new portal vein thrombosis despite ongoing anticoagulation, the possibility of underlying thrombophilia cannot be completely excluded, and previous studies have shown that inherited or acquired thrombophilia can play a significant role in the development of splanchnic vein thrombosis (12, 19). Future studies and case reports should consider including systematic thrombophilia evaluation and long-term follow-up to provide a more comprehensive understanding of risk factors and prognosis in post-transplant splenectomy patients.

## Conclusion

This case report highlights the complexity and challenges associated with managing portal vein thrombosis (PVT) following splenectomy in a post-liver transplant patient. The successful thrombolysis achieved through a combination of anticoagulation therapy and vigilant monitoring underscores several key points:

1. Importance of Adherence to Anticoagulation Therapy: Ensuring patients understand and adhere to their prescribed anticoagulant regimen is crucial to prevent thrombotic complications.
2. Timely Intervention and Monitoring: Regular monitoring and early intervention at the first signs of thrombotic complications can significantly improve patient outcomes, as demonstrated by the positive response to the adjusted anticoagulation regimen and urokinase infusion in this case.
3. Multidisciplinary Care: The management of PVT in liver transplant patients requires a comprehensive, multidisciplinary approach involving hepatologists, surgeons, radiologists, and hematologists to optimize patient care and outcomes.

## Data Availability

The raw data supporting the conclusions of this article will be made available by the authors, without undue reservation.
